# Contemporary Single-Center Experience of Complete Aortic Arch Replacement Employing the Frozen Elephant Trunk Technique in Patients with Extensive Aortic Disease

**DOI:** 10.3390/jcm13226640

**Published:** 2024-11-05

**Authors:** Armin-Kai Schoeberl, Florian Huber, Bruno Schachner, Valentina Preinfalk, Andreas Zierer

**Affiliations:** 1 Department of Cardiothoracic and Vascular Surgery, Kepler University Hospital, Medical Faculty, Johannes Kepler University Linz, 4020 Linz, Austria; 2 Department of Cardiothoracic and Vascular Surgery, Wels-Grieskirchen Hospital, 4600 Wels, Austria

**Keywords:** complete aortic arch replacement, frozen elephant trunk procedure, acute aortic dissection, aortic aneurysm, chronic aortic dissection

## Abstract

**Objective:** This study aimed to examine contemporary results of the frozen elephant trunk (FET) procedure in an all-comers patient cohort. **Methods:** Between January 2017 and May 2024, a total of 132 consecutive patients with either aortic aneurysm (*n* = 32), acute aortic dissection (*n* = 32), or chronic aortic dissection (*n* = 68) underwent total aortic arch replacement employing the FET technique. In-hospital data were collected prospectively and included preoperative characteristics, intraoperative data, and follow-up results. **Results:** The median cardiopulmonary bypass time, cardiac ischemia time, and selective antegrade cerebral perfusion time were 180 (161–205), 89 (70–113), and 45 (38–54) min, respectively. Total 30-day mortality rate was 7.6% (*n* = 10). The rate of major postoperative neurological complications was 6.8% (*n* = 9) for perioperative stroke and 2.3% (*n* = 3) for permanent spinal cord injury. Five patients (3.8%) required hemofiltration at the time of discharge due to postoperative kidney injury. Rates of subsequent endovascular and open aortic repair following primary FET were 40.9% (*n* = 54) and 3.8% (*n* = 5), respectively. The median time to reintervention was 86 (30–439) days. The median follow-up time was 25 (8–52) months, and overall survival rates at 1, 2, and 3 years were 89%, 89%, and 87%, respectively. **Conclusions:** Our data are consistent with current reports, indicating that the FET technique is a valuable adjunct in treating extensive aortic arch pathologies. The procedure provides an increasingly safe and effective option for complete aortic arch replacement, even in patients requiring a redo procedure.

## 1. Introduction

Surgical treatment of complex thoracic aortic aneurysms extending to the aortic arch or descending aorta represents a significant challenge for aortic surgeons. Traditionally, repairing such extensive disease required two separate major procedures involving the replacement of the aortic arch with the deployment of a free-floating prosthesis into the distal aneurysm and thoracoabdominal repair at a later stage [1].

The frozen elephant trunk (FET) procedure, which combines conventional surgery with endovascular techniques, offers a single-stage option to treat patients with extensive thoracic aortic pathologies [2]. Modern hybrid stent grafts allow for complete aortic arch replacement, while the distal end provides an ideal landing zone for secondary interventions. Therefore, the FET procedure is often used as a planned first-stage procedure in the treatment of thoracoabdominal aneurysms [3,4]. Additionally, the indications for this procedure have been extended to include chronic aortic dissection (CAD) and acute aortic dissection (AAD) [5]. While the FET technique is increasingly utilized in various centers, surgical strategies and outcome reports vary considerably [6,7,8]. Herein, we report our single-center experience of 132 patients who underwent the frozen elephant trunk procedure in an all-comers heterogenic group. This study aims to identify trends in short- and mid-term outcomes, focusing on the underlying pathologies and surgical strategies.

## 2. Materials and Methods

Patients undergoing complete aortic arch replacement by deployment of the FET technique between January 2017 and May 2024 were included in this study. In-hospital data were collected prospectively and included preoperative characteristics, intraoperative data, and follow-up results. Outpatient follow-up was conducted according to our institutional protocol and current recommended guidelines, including regular computed tomography scans [5]. This study was approved by the ethics committee of the Kepler University Hospital (IRB-Nr: 1176/2023). Informed consent was waived by the local ethics committee due to the retrospective nature of the study.

### 2.1. Statistical Analysis

Descriptive statistics were used for baseline characteristics. Categorical variables are reported as percentages and counts; continuous variables are reported as median and interquartile range (IQR). A comparison of continuous data between groups was achieved with the analysis of the variance test or Kruskal–Wallis H rank test, as well as the Turkey Kramer pairwise post hoc test or Dunn’s pairwise post hoc test as appropriate. Categorial variables were compared with either the chi-squared test or Fisher’s exact test as appropriate. *p*-values were adjusted using Bonferroni’s method for post hoc tests between study groups. The Kaplan–Meier survival estimate was used to analyze death at follow-up. Log-rank and pairwise testing were used to identify differences in survival between aortic pathologies. *p*-values <0.05 were considered significant. The software SPSS Statistics Version 27.0.1.0 (IBM Corp. 2016., Armonk, NY, USA) was used for statistical analyses.

### 2.2. Indications

**Figure 1:**
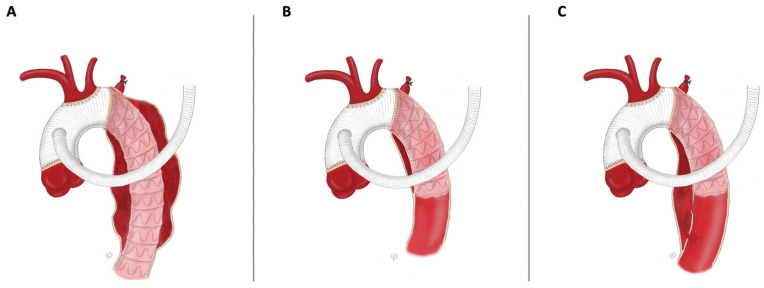
Illustration of the FET procedure as used in complete aortic replacement for extensive aortic pathologies. (**A**) In patients with thoracic aortic aneurysm, (**B**) in patients with acute aortic dissection, and (**C**) in patients with chronic aortic dissection.

Indications for the FET procedure can be divided according to the underlying aortic pathologies. In patients with thoracic aortic aneurysms (TAAs), the FET procedure was used to treat aneurysms involving the aortic arch. In cases with involvement of the descending thoracic aorta, concomitant or subsequent thoracic endovascular aortic repair (TEVAR) was performed. Patients with acute aortic dissection were treated with deployment of the FET technique in cases of type non-A non-B dissection and type A dissections if a tear in the distal arch or proximal descending thoracic aorta (retrograde AAD) was detectable. In a similar fashion, the FET technique was performed for acute type A intramural hematoma or penetrating aortic ulcer if the pathology involved the aortic arch or is in close proximity of the left subclavian artery. Patients with CAD were treated with the FET procedure in cases of dilatation of overall aortic diameter (>55 mm) or dilatation of false lumen with compression of true lumen involving the aortic arch. Illustrations of the FET procedure as used in the treatment of extensive aortic arch disease, divided by the aortic pathology, are available in Figure 1.

### 2.3. Surgical Technique

All patients treated with deployment of the FET technique at our institution were operated, following our standardized institutional surgical and perfusion management protocol. Prior to median sternotomy, arterial cannulation was established via the right axillary artery after systemic heparinization. After cannulation of the right atrial appendage for venous drainage, cardiopulmonary bypass was instituted. Cooling was limited to 28 °C bladder temperature. Myocardial protection was achieved with St. Thomas cardioplegia. The innominate, the left carotid artery, and the left subclavian artery were encircled with silicone elastomer loops and occluded at the beginning of selective antegrade cerebral perfusion. Patients undergoing cardiac surgery at our institution are routinely monitored with transcranial near-infrared spectroscopy (NIRS) to provide continuous information about brain oxygenation. If NIRS measurement dropped below 75% of baseline after unilateral antegrade selective cerebral perfusion (ASCP), bilateral ASCP was immediately established by insertion of a perfusion catheter into the left carotid artery.

After resection of the aortic arch, excluding the island containing the arch vessels, the FET prosthesis was placed into the descending aorta over a guidewire previously placed via the femoral artery. The stented part was then deployed in the descending aorta. The hybrid graft was anastomosed to the proximal descending aorta with 3.0 polypropylene. After the excision of a segment on the cranial part of the not stented prosthesis, the arch vessels were reimplanted en bloc. After completion of the arch replacement, the arch vessels were carefully deaired, and the graft was clamped, followed by reconstitution of full body perfusion. The proximal anastomosis with the native ascending aorta or preexisting grafts was performed during rewarming. Additionally, in cases with a zone 2 distal anastomosis, the left subclavian artery is revascularized with an extra-anatomical aorto-subclavian bypass after reinstating coronary perfusion in order to minimize myocardial ischemia time.

## 3. Results

### 3.1. Baseline Characteristics

Hundred and thirty-two patients underwent complete aortic arch replacement by deployment of the FET technique at our institution over the study period. Three different FET prostheses were used and included the E-vita open (Jotec GmbH, Hechingen, Germany; *n* = 45), the E-vita open NEO (Jotec GmbH, Hechingen, Germany; *n* = 80), and the Thoraflex Hybrid (Vascutek, Terumo, Inchinnan, UK; *n* = 7). Eighty-seven patients were male (65.9%) and 45 female (34.1%), with a median age of 63 years (55–69) at the time of surgery. Thirty-two patients (24.2%) were treated for TAA, 32 patients (24.2%) for AAD, and 68 patients (51.5%) for CAD. Thirty-five patients (25%) had to undergo emergency surgery, while the remaining 97 (75%) underwent an elective procedure. Forty-eight procedures (36.4%) were performed as a redo procedure. Baseline characteristics and preoperative parameters of the 132 subjects are available in Table 1.

### 3.2. Intraoperative Data

Concomitant procedures included aortic valve replacement (*n* = 6), coronary artery bypass grafting (*n* = 10), Bentall procedure (*n* = 5), David procedure (*n* = 1), myectomy (*n* = 1), TEVAR (*n* = 9), and septotomy (*n* = 4). Median cardiopulmonary bypass time, cardiac ischemia time, and ASCP time totaled 180 (161–205), 89 (70–113), and 45 (38–54) min, respectively. Intraoperative data for each group of aortic disease are shown in Table 2.

### 3.3. Early Outcome

The overall 30-day mortality rate, defined as death during initial hospitalization and within 30 days after surgery, was 7.6% (*n* = 10). Reasons for death included myocardial infarction (*n* = 2), fatal stroke (*n* = 3), cerebral bleeding (*n* = 2), cardiogenic shock (*n* = 2), and aortoesophageal fistula (*n* = 1). The majority of these patients presented with AAD (*n* = 7) and required emergency surgery. The overall rate of major postoperative neurological complications, including stroke and permanent postoperative paraplegia due to spinal cord injury, was 6.8% (*n* = 9) and 2.3% (*n* = 3). Perioperative stroke rate was defined as the presence of postoperative lesions in CT/MRI scans and positive neurological evaluation. Transient paraplegia occurred in 6.8% of patients (*n* = 9). Patients showing postoperative symptoms of ischemic spinal injury were treated with cerebrospinal fluid drainage immediately after symptoms were observed. A total of 28 patients (21.2%) required postoperative hemofiltration. Among these cases, we observed a full or partial recovery of kidney function in 23 patients (17.4%), while 5 patients (3.8%) required hemofiltration at the time of discharge. Postoperative results divided by each group of underlying aortic disease and overall population are available in Table 3. When comparing perioperative outcome data between male (*n* = 87) and female (*n* = 45) patients, we observed a significant difference in the median ICU stay favoring female patients (*p* = 0.023). Aside from this, no gender-specific differences in postoperative complications and outcomes were found. Gender-specific outcome data are available in Supplementary Table S1.

### 3.4. Follow-Up

**Figure 2:**
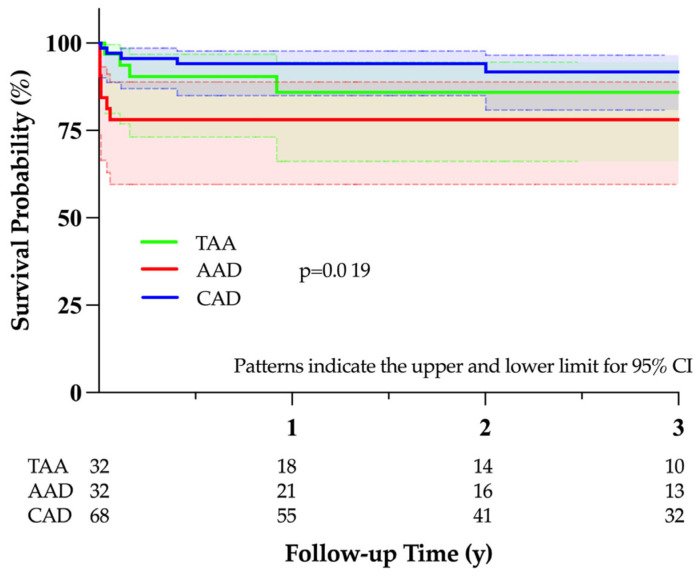
Kaplan–Meier survival probability of each group of aortic disease. TAA, thoracic aortic aneurysm; AAD, acute aortic dissection; CAD, chronic aortic dissection; CI, confidence interval.

Median follow-up time was 25 (8–52) months. Overall survival rates at 1, 2, and 3 years were 89%, 89%, and 87%, respectively. The Kaplan–Meier survival probability of each group of aortic disease is available in Figure 2. AAD was associated with a significantly reduced survival probability, potentially caused by the high perioperative mortality (*p* = 0.019). In comparing postoperative survival between male (*n* = 87) and female (*n* = 45) patients, no significant difference was observed between the groups (*p* = 0.512).

### 3.5. Secondary Aortic Interventions

A total of 54 (40.9%) patients required endovascular repair of the downstream aorta after the primary FET procedure. Twenty-seven aortic reinterventions (20.5%) were performed as planned staged procedures, while the rest were performed after the indication was confirmed on a follow-up CT scan. Indications included diameter progression of the downstream aorta (*n* = 17), distal stent-induced neo entry (*n* = 4), collapsing true lumen (*n* = 4), kinking of the stent graft (*n* = 1), and type III endoleak (*n* = 1). Only five patients (3.8%) required open aortic surgical repair after the FET procedure. Reasons for open aortic reoperation included infected FET prosthesis (*n* = 1), pseudoaneurysm of the distal or proximal anastomosis (*n* = 3), and open thoracoabdominal repair due to progression of the thoracoabdominal aneurysm (*n* = 1). The median time to reintervention was 86 (30–439) days. 

## 4. Discussion

Although various outcome reports of the frozen elephant trunk procedure are available, most analyses are conducted over an extended study period. Since experience with this hybrid procedure has increased significantly in the last decade, contemporary reports can provide insights into the changing landscape of the frozen elephant trunk procedure and complete aortic arch replacement. Early reports of the FET procedure were associated with high rates of postoperative mortality, neurological dysfunction, and kidney injury [6,7,8,9,10,11]. However, contemporary results of the FET technique indicate a satisfactory trend with decreased rates of perioperative mortality (1.8–14%), stroke (1.8–18.2%), SCI (0–5%), permanent postoperative kidney injury (3–4.5%), and rethoracotomy for bleeding (3–14%) [7,8,12,13,14,15]. Our results further support the increasingly favorable outcome data of patients treated with the FET technique for various underlying aortic pathologies.

Although patients undergoing surgery for CAD were predominantly redo procedures (55.9%), the 30-day mortality rate was lowest among the subgroups at 2.9% (*n* = 2). This further suggests that the FET procedure can be safely performed in patients after previous aortic repair [16,17]. The constant refinement of surgical management, technical advances, and growing experience with this hybrid procedure have made a considerable contribution to the increasingly satisfying results of the FET procedure. Newer stent grafts offer various vascular configurations, including straight, branched, and trifurcated, therefore improving individual surgical management. Additionally, newer stent grafts are shorter in length, which is potentially associated with the decreasing rate of SCI [18]. In this patient cohort, we predominantly used straight vascular grafts with subsequent island implantation of the arch vessels. This surgical approach requires only two vascular anastomoses in hypothermic circulatory arrest and can minimize ASCP time. In cases with Zone 2 distal anastomosis, we perform an extra-anatomical aorto-subclavian bypass during reperfusion and rewarming after opening the aortic clamp. This option can offer a same-stage, minimally invasive option of revascularization without prolonging cardiopulmonary bypass time. The proximalization of the distal anastomosis to Zone 2 allows for a technically easier operation and can therefore improve patient outcome [19,20,21]. Another major advancement of open surgical aortic arch repair in recent years is the utilization of unilateral or bilateral ASCP, which consequently makes deep hypothermia nonessential. Therefore, there is a growing tendency to increase body temperature during circulatory arrest, which can decrease hypothermia-related side effects [22,23].

Despite the increasing experience and advancing surgical techniques, the use of the FET procedure in patients with acute aortic dissection remains controversial. Although some reports indicate promising results in patients with AAD undergoing the FET procedure with mortality rates as low as 1–12% [13,15,24], the 30-day mortality rate in our series remained high at 21.9%. This can potentially be explained by our reservations about deploying the FET technique in patients with AAD if not absolutely indicated by the anatomic conditions or distal malperfusion. Patients presenting with acute aortic dissection requiring emergency surgery are treated with an initial proximal aortic repair and potential complete aortic arch replacement with the FET technique at a later stage [25]. This is due to our opinion that patients in an emergency setting benefit from a limited repair, whereas the FET procedure can safely be performed as a reoperation. Therefore, patients in this cohort with AAD who required an extended repair with a primary FET procedure presented with an entry tear in the aortic arch, the descending aorta (retrograde AAD), or significant distal malperfusion, which is associated with a worse perioperative outcome [26]. 

While surgical repair remains the first-line therapy for patients with aortic disease involving the aortic arch, endovascular arch reconstruction is increasingly utilized [27]. In these cases, intentional coverage of the left subclavian artery or carotid arteries is often performed to ensure an adequate proximal landing zone, which frequently necessitates routine revascularization [28]. However, newer custom-made prostheses, using in-situ fenestrations or single-branch designs, can offer a less invasive, same-stage option for endovascular repair involving the aortic arch in such patients [29,30]. Endovascular extension of the downstream aorta following the FET procedure was performed in 54 patients (40.9%), while only 5 patients required open surgical aortic repair due to FET-related complications such as pseudoaneurysm of the distal or proximal anastomosis. Compared to other reports, our rate of elective endovascular extensions is considerably higher. Potential reasons include our aspirations to entirely treat segments of the diseased thoracoabdominal aorta in patients with TAA or stabilize the downstream aorta in patients with enlargement of the false lumen or distal aorta. This is reflected by the high rate of planned staged procedures in this patient cohort. While open surgery of the thoracoabdominal aorta after the FET procedure can be performed with acceptable results [31], many centers have adopted a completely endovascular approach to treat pathological segments of the downstream aorta [32,33]. At our center, we follow a similar approach by extending the stent graft down to the level of the iliac vessels if indicated. In cases of collapsing true lumen or takeoff of aortic branches from the false lumen, we perform extensive thoracoabdominal electro-aortic septotomy. This procedure creates a new common lumen, facilitating subsequent total endovascular repair in patients otherwise only treatable with high-risk surgical thoracoabdominal aortic replacement [34]. Planned staged procedures are performed 6 weeks after the primary FET procedure to ensure collateral network development for spinal cord perfusion. 

The following limitation has to be discussed: the retrospective design of this study might confer a selection bias and limit the quality of the retrieved data. Strengths of this study include a uniform surgical treatment approach and a high number of patients treated in a relatively short study period. In conclusion, due to the continued refinement of surgical techniques and growing experience, the FET procedure offers an increasingly reliable safety profile for complete aortic arch repair, even when performed as a redo surgery. Additionally, in patients with progressive aortic disease, the distal portion of the FET prosthesis serves as a secure landing zone for future endovascular aortic interventions.

## Figures and Tables

**Table 1:** Preoperative characteristics.

Preoperative Characteristics	TAA (*n* = 32)	AAD (*n* = 32)	CAD (*n* = 68)	Total (*n* = 132)	*p*
Age, y	68 (62–73)	62 (51–69)	60 (53–67)	63 (55–69)	0.005 *^†^
Male	18 (56.3%)	22 (68.8%)	47 (69.1%)	87 (65.9%)	0.416
Height	170 (162–178)	175 (169–182)	175 (168–180)	174 (168–180)	0.053
Weight	71 (61–178)	83 (72–98)	81 (73–96)	80 (70–95)	0.030 *
Heart failure	4 (12.5%)	1 (3.1%)	1 (1.5%)	6 (4.5%)	0.047
COPD	6 (18.8%)	1 (3.1%)	5 (7.4%)	12 (9.1%)	0.115
Hypertension	20 (62.5%)	19 (59.4%)	48 (70.6%)	87 (65.9%)	0.647
Chronic kidney disease	9 (28.1%)	3 (9.4%)	11 (16.2%)	23 (17.4%)	0.156
Preoperative dialysis	1 (3.1%)	0	2 (2.9%)	3 (2.3%)	0.999
Peripheral arterial disease	4 (12.5%)	0	1 (1.5%)	5 (3.8%)	0.037
Cerebrovascular disease	2 (6.3%)	1 (3,1%)	1 (1.5%)	4 (3.0%)	0.434
Diabetes	2 (6.3%)	2 (6.3%)	5 (7.4%)	9 (6.8%)	0.999
Redo-Procedure	7 (21.9%)	3 (9.4%)	38 (55.9%)	48 (36.4%)	<0.001 ^†‡^

* *p*-value < 0.05 TAA versus AAD in post hoc test. ^†^ *p*-value < 0.05 TAA versus CAD in post hoc test. ^‡^ *p*-value < 0.05 AAD versus CAD in post hoc test. TAA, thoracic aortic aneurysm; AAD, acute aortic aneurysm; CAD, chronic aortic aneurysm; COPD, chronic obstructive pulmonary disease.

**Table 2:** Intraoperative data.

Intraoperative Data	TAA (*n* = 32)	AAD (*n* = 32)	CAD (*n* = 68)	Total (*n* = 132)	*p*
Procedure time, min	337 (299–355)	344 (313–404)	332 (282–376)	336 (300–373)	0.145
Cardiopulmonary bypass time, min	188 (144–204)	190 (179–223)	175 (155–200)	180 (161–205)	0.022 ^‡^
Cardiac ischemia time, min	83 (71–117)	109 (90–123)	78 (66–102)	89 (70–113)	0.001 *^‡^
ASCP time, min	42 (35–54)	50 (41–56)	43 (38–54)	45 (38–54)	0.099
ASCP number					
Unilateral	10 (31.3%)	19 (59.4%)	30 (44.1%)	59 (44.7%)	
Bilateral	22 (68.8%)	13 (40.6%)	38 (55.9%)	73 (55.3%)	
Distal Anastomosis					
Zone 2	15 (46.9%)	19 (59.4%)	38 (55.9%)	72 (54.5%)	
Zone 3	17 (53.1%)	13 (40.6%)	13 (40.6%)	60 (45.5%)	
Concomitant Procedure Aortic valve replacement Bentall procedure David procedure CABG TEVAR: Myectomy Septotomy	12 (37.5%) 3 (9.4%) 1 (3.1%) 0 7 (21.9%) 1 (3.1%) 0 0	7 (21.9%) 0 3 (9.4%) 0 1 (3.1%) 1 (3.1%) 1 (3.1%) 1 (3.1%)	17 (25%) 3 (4.4%) 1 (1.5%) 1 (1.5%) 2 (2.9%) 7 (10.3%) 0 3 (4.4%)	36 (27.3%) 6 (4.5%) 5 (3.8%) 1 (0.8%) 10 (7.6) 9 (6.8%) 1 (0.8%) 4 (3.0%)	0.195

* *p*-value < 0.05 TAA versus AAD in post hoc test. ^‡^ *p*-value < 0.05 AAD versus CAD in post hoc test. TAA, thoracic aortic aneurysm; AAD, acute aortic aneurysm; CAD, chronic aortic aneurysm; ASCP, antegrade selective cerebral perfusion; CABG, coronary artery bypass grafting; TEVAR, thoracic endovascular aortic repair.

**Table 3:** Postoperative complications and outcome.

Postoperative Data	TAA (*n* = 32)	AAD (*n* = 32)	CAD (*n* = 68)	Total (*n* = 132)	*p*
Stroke	1 (3.1%)	4 (12.5%)	4 (5.9%)	9 (6.8%)	0.389
Spinal cord injury					
Transient	3 (9.4%)	2 (6.3%)	4 (5.9%)	9 (6.8%)	0.898
Permanent	1 (3.1%)	1 (3.1%)	1 (1.5%)	3 (2.3%)	0.611
Hemodialysis					
Transient	7 (21.9%)	10 (31.3%)	6 (8.8%)	23 (17.4%)	0.016 ^‡^
Permanent	1 (3.1%)	3 (9.4%)	1 (1.5%)	5 (3.8%)	0.106
Prolonged ventilation (>72 h)	3 (9.4%)	10 (31.3%)	2 (2.9%)	15 (11.4%)	<0.001 ^‡^
Rethoracotomy	0	6 (18.8%)	6 (8.8%)	12 (9.1%)	0.034 *
ICU median stay, d	4 (2–6)	6 (3–10)	4 (2–6)	4 (2–7)	0.042 ^‡^
Hospital median stay, d	21 (12–35)	16 (10–33)	21 (15–27)	21 (13–30)	0.732
Endovascular Reintervention	15 (46.9%)	9 (28.1%)	30 (44.1%)	54 (40.9%)	0.232
In-hospital mortality (30 d)	1 (3.1%)	7 (21.9%)	2 (2.9%)	10 (7.6%)	0.004 ^‡^

* *p*-value < 0.05 TAA versus AAD in post hoc test. ^‡^ *p*-value < 0.05 AAD versus CAD in post hoc test. TAA, thoracic aortic aneurysm; AAD, acute aortic aneurysm; CAD, chronic aortic aneurysm; ICU, intensive care unit.

## Data Availability

Data are available from the corresponding author upon reasonable request.

## Supplementary Materials

The following supporting information can be downloaded at: https://www.mdpi.com/article/10.3390/jcm13226640/s1, Table S1: Gender-specific outcome data.
