# Supplementary material for: Contemporary Single-Center Experience of Complete Aortic Arch Replacement Employing the Frozen Elephant Trunk Technique in Patients with Extensive Aortic Disease
Source: J Clin Med. 2024 Nov 5;13(22):6640. doi: 10.3390/jcm13226640 (PMC11595266; doi:10.3390/jcm13226640)
Supplement: Supplementary file 1 [file jcm-13-06640-s001.zip › jcm-3271376-supplementary.pdf]

Postoperative data	Male (n=87)	Female (n=45)	Total (n=132)	P
Stroke	7 (8.0%)	2 (4.4%)	9 (6.8%)	0.718
Spinal cord injury				
Transient	6 (6.9%)	3 (6.7%)	9 (6.8%)	0.634
Permanent	3 (3.5%)	0	3 (2.3%)	0.551
Haemodialysis				
Transient	18 (20.7%)	5 (11.1%)	23 (17.4%)	0.185
Permanent	4 (4.6%)	1 (2.2%)	5 (3.8%)	0.300
Prolonged ventilation (>72h)	12 (13.8%)	3 (6.7%)	15 (11.4%)	0.214
Rethoracotomy	8 (9.2%)	4 (8.9%)	12 (9.1%)	0.613
ICU median stay, d	4 (3-7)	3 (2-6)	4 (2-7)	0.023
Hospital median stay, d	20 (12-31)	21 (15-30)	21 (13-30)	0.520
Endovascular Reintervention	36 (41.4%)	18 (40%)	54 (40.9%)	0.232
In-hospital mortality (30d)	7 (8.0%)	3 (6.7%)	10 (7.6%)	0.748
